# Superior mesenteric artery dissection with prolonged abdominal angina treated by laparotomy, endarterectomy, patch angioplasty, and retrograde open mesenteric stenting: a case report

**DOI:** 10.1186/s40792-019-0736-0

**Published:** 2019-10-29

**Authors:** Shinichi Tanaka, Atsushi Fukuda, Eisuke Kawakubo, Takuya Matsumoto

**Affiliations:** 1 Division of Vascular Surgery, Saiseikai Karatsu Hospital, 817 Motohata-machi, Karatsu city, Saga 847-0852 Japan; 2 Department of Surgery and Science, Graduate School of Medical Sciences, Kyushu University, 3-1-1 Maidashi, Fukuoka, Higashi-ku 812-8582 Japan; 3 Department of Vascular Surgery, Graduate School of Medical Sciences, International University of Health and Welfare, 4-3 Kouzunomori, Narita, Chiba 286-8686 Japan

**Keywords:** Superior mesenteric artery, Dissection, Thromboendarterectomy, Stent

## Abstract

**Background:**

Most patients with isolated superior mesenteric artery (SMA) dissection are successfully managed conservatively. However, some patients require more invasive treatment.

**Case presentation:**

We herein describe a 45-year-old man with isolated SMA dissection. He initially underwent conservative treatment. However, because of persistent abdominal angina, we considered the need for surgical revascularization. He was successfully treated by endarterectomy, patch angioplasty, and retrograde open mesenteric stenting. The abdominal angina was stabilized thereafter.

**Conclusions:**

The combination of endarterectomy, patch angioplasty, and retrograde open mesenteric stenting is useful for isolated SMA dissection, and long patency can be expected for some patients.

## Background

Superior mesenteric artery (SMA) dissection was historically thought to be rare but has been identified more frequently with the development of imaging techniques. The etiology of SMA dissection remains unclear, and the optimal management technique for treatment has not been established [1–4]. Almost all patients are successfully managed conservatively; however, some patients require more invasive treatment. Bowel resection, thromboendarterectomy, stenting, and surgical bypass have been reported, but few reports have provided details regarding the indications for treatment.

We herein report a case of acute SMA dissection without intestinal ischemic necrosis but with persistent abdominal angina. The patient was successfully treated by laparotomy, thromboendarterectomy, and retrograde stenting.

## Case presentation

**Fig. 1:**
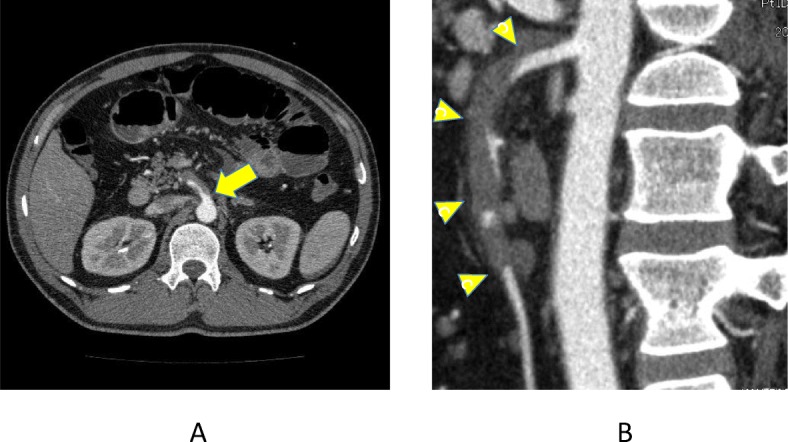
Computed tomography images 4 days after the symptom onset. **a** The main trunk of the superior mesenteric artery was dissected from its origin (arrow). **b** Sagittal multiplanar reconstruction computed tomography showed the thrombosed false lumen and compressed true lumen with thrombosis at the distal portion (arrowheads) and enhancement at the more distal portion

A 45-year-old man experienced abdominal pain and vomiting. He consulted a nearby doctor, who prescribed an intestinal preparation. The patient developed ten episodes of diarrhea the next day and received infusion therapy. He experienced a persistent sense of abdominal fullness and left abdominal pain for 3 days and then consulted our hospital. The patient was previously healthy and had no remarkable medical history or cardiovascular risk factors. He had no smoking habit. Physical examination revealed mild tenderness over the epigastrium without signs of peritonitis. Enhanced computed tomography (CT) revealed an isolated dissection of the SMA in which the false lumen was thrombosed, the true lumen was compressed by the false lumen, and the middle colic artery to small colon branches were obstructed (Fig. 1). No signs of bowel ischemia, such as bowel thickening, abnormal contrast enhancement, or ascites, were found. The creatine phosphokinase level, aspartate aminotransferase level, lactate dehydrogenase level, and base excess were normal at 210 IU/L, 22 U/L, 189 U/L, and 0.3 mmol/L, respectively, at the first visit to our hospital (3 days after onset). The lactate level was not measured at our hospital, but there was a mention in the medical information offer letter that it was normal at a university hospital.

**Fig. 2:**
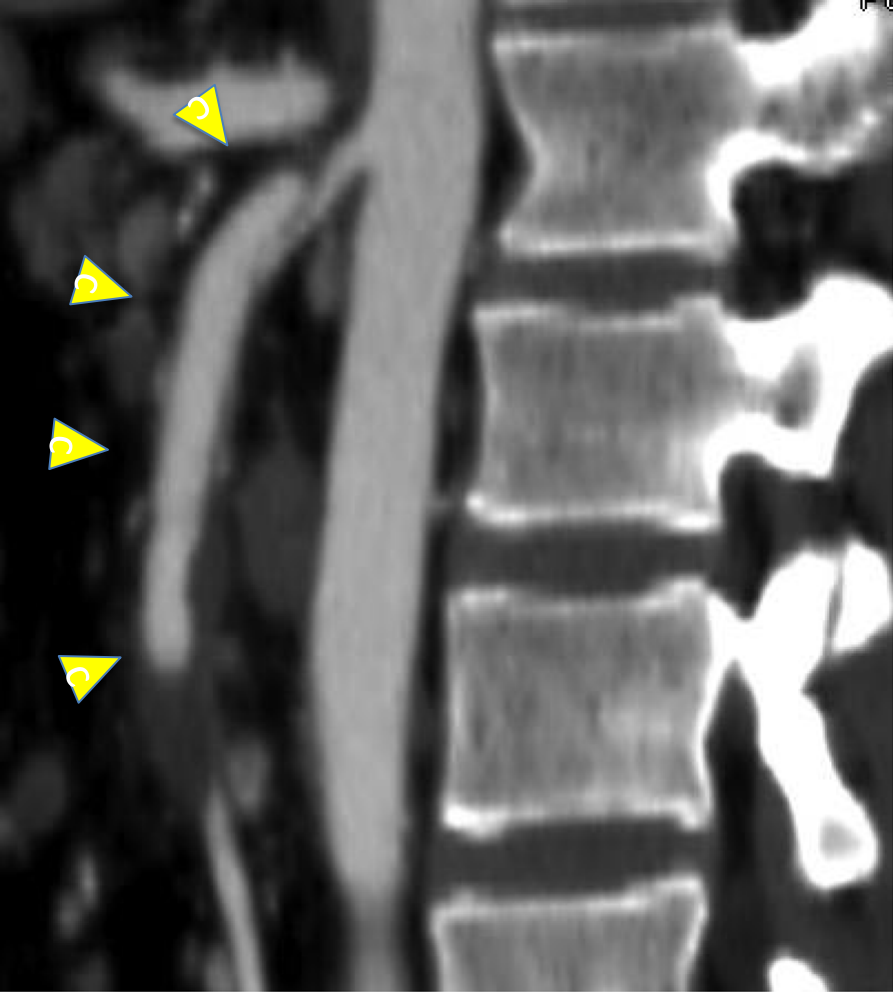
Computed tomography image 36 days after the symptom onset. Sagittal multiplanar reconstruction computed tomography showed that the false lumen was enhanced (arrowheads), but the true lumen was still compressed and thrombosed at the distal portion

Because of the patient’s continuous abdominal pain, we considered the need for emergency surgical therapy and transferred him to our university hospital. He was treated by conservative therapy including antiplatelet agents and prostaglandin, and his continuous abdominal pain disappeared. He was discharged on day 20 and returned to our hospital for follow-up. We performed nutritional management involving central venous nutrition followed by oral intake. We changed his liquid diet to rice gruel, but his nutritional state and oral intake did not improve. On day 35, enhanced CT showed that the area around the SMA was hypodense with abnormal contrast enhancement, and severe stenosis of the true lumen and a 3-cm-long thrombotic occlusion from the middle colic artery were observed (Fig. 2).

We performed a surgical operation on day 40 because of the prolonged abdominal angina. Spontaneous abdominal pain and tenderness had been present at the first visit, but these symptoms disappeared before the operation.

**Fig. 3:**
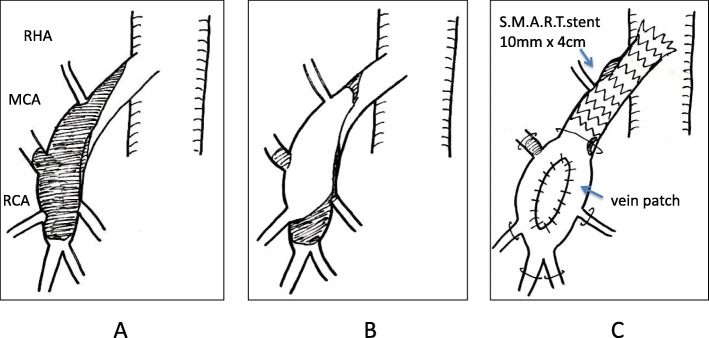
Schema of proximal superior mesenteric artery portion **a** 4 days after the symptom onset, **b** 36 days after the symptom onset (before revascularization), and **c** after revascularization. RHA, right hepatic artery; RCA, right colic artery; MCA, middle colic artery

The SMA was revascularized through a midline abdominal incision. During laparotomy, the transverse colon was pulled toward the head side, the origin of the small intestinal mesentery was divided, and the SMA was exposed and controlled at the branch of the middle colic artery. First, we performed thromboendarterectomy. We longitudinally incised the SMA from the thrombotic occluded part to the false lumen, performed thromboendarterectomy with a flap under a direct view, inserted a sheath into the central side (probably the false lumen), clamped the site with vascular tape, and placed an endovascular stent on the central side. A short 0.035-in. guidewire was introduced, and a 6-Fr sheath was placed in a retrograde fashion. Hand-injected retrograde angiography was performed; the false lumen and SMA orifice exhibited contrast enhancement, but the true lumen did not. A 0.035-in. guidewire was used to cross the true lumen into the aorta. The SMA then showed a stenotic area by contrast enhancement. A 10-mm × 4 cm stent (S.M.A.R.T.; Cordis Corp., Fremont, CA, USA) was placed in a retrograde manner at the SMA origin (Fig. 3). We then performed a patch angioplasty using the great saphenous vein.

**Fig. 4:**
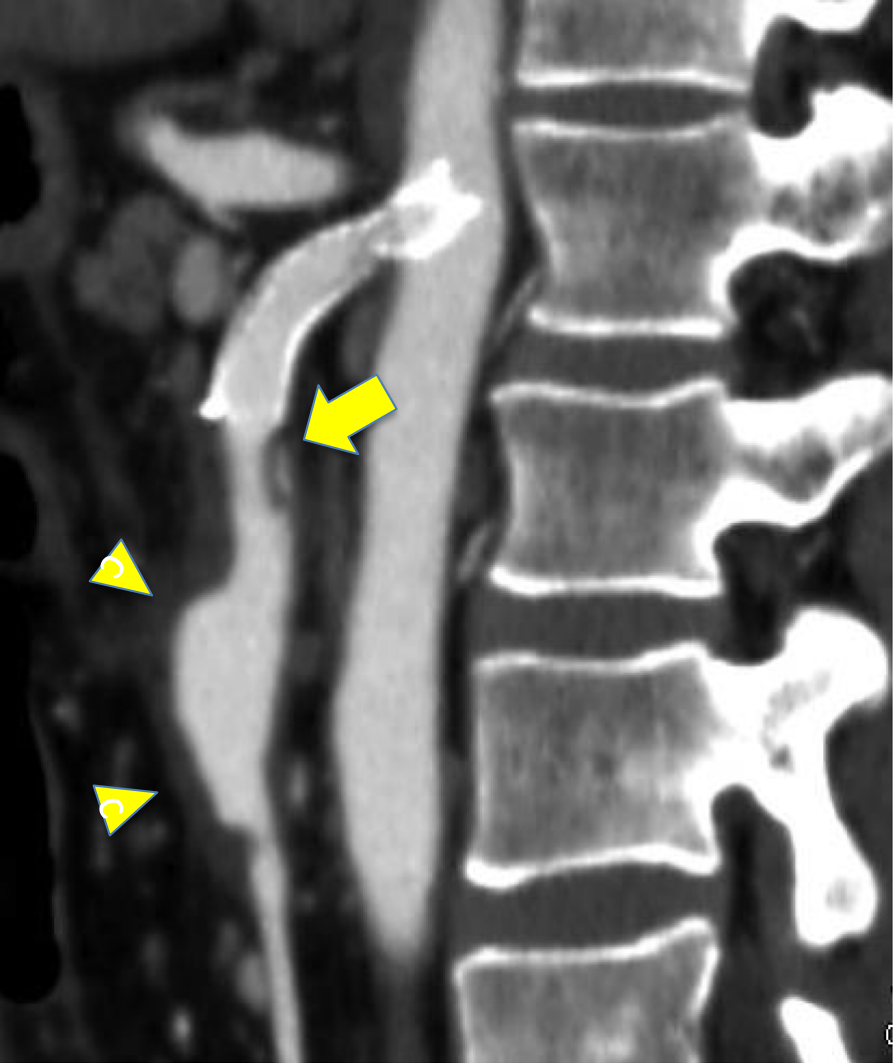
Computed tomography image at 91 days postoperatively. The true lumen of the superior mesenteric artery was dilated at the patch angioplasty site (arrowheads), the stent was patent, and a small area lacking contrast medium was present at the remaining flap (arrow)

The pulse of the distal SMA then improved. Three days after the operation, oral intake was started and the abdominal angina stabilized. The patient was discharged 26 days after the operation. At 58 days after the operation, he was hospitalized again because of hypoalbuminemia and whole body edema. Lower digestive tract endoscopy showed no mucosal disorders, and CT showed a patent stent and no signs of bowel ischemia. We considered that the patient’s symptoms had arisen from a disorder of the absorptive surface area due to chronic weak bowel ischemia and long-term fasting. Central venous nutrition was initiated, and his frequency of diarrhea decreased. Oral intake was then begun, and as his oral intake increased, his nutritional status improved. He was discharged 28 days after readmission. Follow-up CT showed good flow and a thin flap between the stent and patch (Fig. 4). He was symptom-free 3 years after the surgery with a good nutritional status.

## Discussion

Isolated dissection of a splanchnic artery is a rare condition, and most reports of splanchnic artery dissection involve the SMA [1–4].

Sakamoto et al. [5] categorized SMA dissection into four types based on contrast-enhanced CT findings. The present case was diagnosed as type IV (completely thrombosed false lumen without an ulcer-like projection). When bowel ischemia is present in the acute stage, emergency surgery is indicated. Otherwise, patients undergo conservative medical treatment or regular imaging surveillance and comprehensive follow-up [1, 2, 6, 7]. In the present case, the persistent and worsening symptoms of abdominal angina despite anticoagulation or antiplatelet therapy indicated the need for surgery.

After our patient’s operation, the bowel ischemia improved; however, his diarrhea and disorder of absorption persisted and his serum albumin remained low. Surgical revascularization and stent placement should be performed in patients with evidence of bowel ischemia, such as poor contrast enhancement, edematous thickening, true lumen thrombosis or stenosis, disturbed peripheral flow, or poor collateral flow. It takes time to recover from the mucosal damage caused by bowel ischemia.

It is important to shift to surgical treatment without delay when the patient’s condition is not improved by conservative treatment. The main types of surgical revascularizations reported in the literature are thromboendarterectomy, stenting, and bypass (Table 1).

**Table 1:** Reported representative open treatment

Method		Age/sex	Bowel resection
Thrombectomy	Shigemitsu et al. [6]	62/M	No
		65/M	Yes
Thrombectomy and iliac-ileal bypass	Satokawa et al. [1]	53/M	No
		47/M	No
Iliac-distal SMA bypass	Katsura et al. [7]	50/M	No
		46/F	No
ROMS	Matushita et al. [8]	78/M	No
		58/M	No
	Wyers et al. [9]	60/M	Yes
		66/F	No
		67/F	Yes
		68/F	No
		62/F	No
		52/M	Yes

Because of the risk of bending of the autologous vein bypass and the risk of low patency and infection of the artificial blood vessels, we did not choose bypass for revascularization. If the extent of the dissection is long, as in patients undergoing thromboendarterectomy alone, it may be difficult to obtain a visual field that exposes the central dissection under direct vision. We think that it is dangerous that the central flap is blindly removed. Cannulation to the true lumen or complete thrombolysis is thought to be difficult to achieve by percutaneous transcatheter therapy using a brachial or femoral approach because dissection occurs near the SMA origin. In the present case, there was a large difference in the diameter between the area of dissection, occlusion, and peripheral opening; therefore, we performed both thromboendarterectomy and stenting.

Matsushita et al. [8] recently described two patients treated with retrograde open mesenteric stenting (ROMS). Neither a plain balloon nor stenting only is adequate because of the difference between the diameter of the peripheral and central site. Placing the sheath near the occlusion site makes it easy to cannulate the lesion, directly observe the bowel viability, and achieve anatomic revascularization. Wyers et al. [9] stated that because this technique is combined with open laparotomy, it honors the essential surgical principles of evaluating and resecting nonviable bowel. Additionally, there is no risk of graft flexion or other problems by bypass surgery. Roussel et al. [10] reported a 91% primary patency rate among patients who underwent retrograde open mesenteric stenting for acute thrombotic mesenteric ischemia. Thus, adequate long-term patency can be expected.

The patched area was considerably larger than the original blood vessel diameter in the present case. A smaller patch may have been adequate. The requirement for surgical revascularization should be determined on a case-by-case basis. If a large difference in diameter exists between the area of dissection, occlusion, and peripheral opening site, it may be useful to perform endarterectomy with patch angioplasty of the obstructed area and retrograde stenting.

## Conclusions

In summary, we have herein reported a case of isolated SMA dissection with prolonged abdominal angina that was treated by laparotomy, endarterectomy, patch angioplasty, and retrograde open mesenteric stenting. This method is a useful technique for patients with SMA dissection and bowel ischemia when performed in a proper endovascular therapy room, such as a hybrid operation room.

## Data Availability

Not applicable.

## Abbreviations

CT: Computed tomography
ROMS: Retrograde open mesenteric stenting
SMA: Superior mesenteric artery
